# Initial performance evaluation of the UniCel® DxH 800 Coulter® cellular analysis system

**DOI:** 10.1111/j.1751-553X.2010.01239.x

**Published:** 2011-02

**Authors:** B D HEDLEY, M KEENEY, I CHIN-YEE, W BROWN

**Affiliations:** Department of Hematology, London Health Sciences CentreLondon, ON, Canada

**Keywords:** Hematology analyzer, WBC, NRBC, platelet count, extreme leukocytosis, interference

## Abstract

The Beckman Coulter UniCel® DxH 800 is a hematology analyzer incorporating new electronic and mechanical design with advanced algorithm technology to perform CBC, white blood cell (WBC) differential, nucleated red blood cell (NRBC), and reticulocyte analysis. Evaluation of this instrument was performed in our 800-bed tertiary care hospital and specifically centered upon the correlation of WBC, NRBC, and platelet (PLT) enumeration when compared to a predicate analyzer, the Coulter® LH 780, and flow cytometry (FCM) reference methods. Of particular interest were those samples with morphologically confirmed interference and extreme leukocytosis (evaluated with respect to red blood cell parameter correction). The sample set (*n* = 272) consisted of morphologically normal and hematologically abnormal patients. Correlation of the WBC, PLT, and NRBC showed *r*^2^ values of 0.994, 0.985, and 0.910 for the DxH 800 *vs*. FCM, respectively. The presence of interfering particles did not affect the accuracy of the DxH 800 with respect to WBC counts. The DxH 800 showed accurate PLT and NRBC counts in the clinically significant low range when compared to FCM. Compared to the LH 780, flagging rates were significantly reduced (NRBC flag), or equivalent (WBC, PLT flag) on the DxH 800. The DxH 800 demonstrated higher sensitivity and specificity for PLTs and NRBCs and achieved a lower NRBC false negative rate compared to the LH 780. The UniCel® DxH 800 represents a significant improvement to previous impedance analyzers in accurately detecting the presence of NRBCs at counts >1/100 WBC. Furthermore, it provides accurate PLT and WBC counts in the presence of interference and improved NRBC flagging efficiency when compared to the LH 780. Correction of red blood cell parameters is appropriate and accurate in cases of extreme leukocytosis.

## Introduction

The accurate automated enumeration and identification of platelets (PLTs), white blood cells (WBCs), and nucleated red blood cells (NRBCs) is an important and often challenging aspect of the complete blood count (CBC). Confirmation of accuracy of any of these parameters is time-consuming, costly, and the delay in reporting may have a negative impact on patient management. The new Beckman Coulter UniCel® DxH 800 Coulter® Cellular Analysis System (DxH 800; Miami, FL, USA) complete blood cell counter combines advanced hardware technology, innovative computer algorithms, impedance technology, flow cell volume, conductivity, and five light scatter measurements to analyze PLTs, WBCs, and NRBCs. These technologies integrate to define a new and more accurate ‘signature position’ occupied by NRBCs as well as to identify discrete WBC populations and common cellular interferences. This data analysis is performed as part of every routine CBC and WBC differential without the need for additional reagent.

Highly accurate automated WBC, PLT, and NRBC counts, especially in the presence of interference, has a large impact in the operational efficiency of the hematology laboratory (Schwartz & Stansbury, 1954; Fernandez *et al.*, 2001; La Porta, Bowden & Barr, 2002; Bourner, Dhaliwal & Sumner, 2005; Harrison *et al.*, 2005; Segal *et al.*, 2005; Bodey, 2009). The presence of interfering particles in automated blood cell enumeration often triggers a ‘flag’ for manual review. Although flags are helpful and results in film review, this flagging is often not sensitive enough (resulting in false negatives) or misidentifies other cellular types (resulting in false positives) (Brown *et al.*, 2001; Chin-Yee *et al.*, 2001, 2002; Keeney, Brown & Chin-Yee, 2001; Muller *et al.*, 2006; Ghys, Malfait & J, 2009). Inaccurate automated enumeration of any parameter involves the traditional labor-intensive process of manually inspecting and assessing a peripheral blood film for each of these parameters. Therefore, technologies that refine automated cell counting so that flagging rates are reduced (by increasing specificity and sensitivity) to those samples which absolutely require manual inspection have the potential to significantly improve workload and turn around times. The goal of this study was to evaluate the new Beckman Coulter UniCel® DxH 800 cellular analysis system centered upon correlation of WBC, NRBC, and PLT enumeration when compared to a predicate analyzer, the Coulter® LH 780, and flow cytometry (FCM) reference methods.

The protocols and methodologies used in this evaluation were those of the International Committee for Standardization in Hematology (ICSH) and the Clinical Laboratory Standards Institute (CLSI) for reference differential counts and the evaluation of instrument methods (ISLH 1994; Richardson Jones *et al.*, 1996; Koepke *et al.*, 2007).

## Materials and methods

Evaluation and validation of the DxH 800 cellular analysis system (with software version 1.0.0) was carried out in our hospital laboratory, an 800-bed tertiary care hospital with over 700 000 inpatient, outpatient, and emergency visits annually. The hospital provides services for a large region and includes specialized services such as critical care/trauma, cardiology, neurology, cancer care, and transplant (solid and hematopoietic) medicine.

## Specimens

A total of 272 specimens (including morphologically normal and hematological abnormal specimens) were analyzed. All samples were anticoagulated with K_2_EDTA and were spent routine hematology clinical samples. Samples were stored at room temperature and tested within 8 h of collection. All samples were run initially on the LH 780 in the CDR mode (primary aspiration), followed by similar testing on the comparator instrument (DxH 800). WBC, NRBC, and PLT counts were performed on the entire sample set using previously published FCM reference methods (Chin-Yee *et al.*, 2001, 2002; Kaplan, Johnson & Wolfe, 2001; Keeney, Brown & Chin-Yee, 2001). The samples recruited for the study were specifically chosen so that the analytical range of the DxH 800 was challenged. All samples were morphologically examined and only those found to have NRBCs, giant PLTs, or PLT clumping on a blood film were considered truly positive for those abnormalities.

## DxH 800

The DxH 800 is a fully automated analyzer that provides a CBC, WBC differential, reticulocyte percentage and number, and NRBC enumeration. Advanced signal to noise algorithms have been developed with the goal of improving the reportable range of this new instrument (see Table 1), especially in the presence of interference. The system comprises a data management module running on Microsoft™ Windows XP™ which can store a database of up to 40 000 patient results with graphical representation via a touch screen. Overall, the DxH 800 cellular analysis system has a smaller footprint that the predicate LH 780 analyzer. A digital bar code reader with 2D bar code capability is used for loading control materials and reagents which should help in reducing errors in instrument setup and accurately tracking reagent use. The capacity is twenty separate five-tube cassettes with a maximal automated throughput of 100 samples per hour. The aspiration volume in both open/closed vial modes has been reduced to 165 μl of sample allowing for small volume samples to be run (e.g. pediatric samples). A new flow cell design supports additional light scatter measurements enabling enhanced data acquisition for the WBC differential, reticulocyte, and NRBC analyses. This enhanced data collection allows the DxH 800 to collect 10 times more information on each sample when compared to predicate analyzers. These advances allow the DxH 800 to accurately determine a WBC count even in the presence of interference. A new feature on the DxH 800 is the appropriate correction of RBC parameters in the presence of leukocytosis [uncorrected (UWBC) ≥11 × 10^9^/l]. Hemoglobin is corrected when the UWBC count is ≥11 × 10^9^/l and if the UWBC count is ≥140 × 10^9^/l then the RBC count is automatically corrected. The MCV, red cell distribution width (RDW), and red cell distribution width standard deviation (RDWSD) are corrected only if there is evidence that the WBC events are negatively impacting the RBC histogram. The accuracy of the DxH 800 was determined by analyzing samples on a predicate analyzer (LH 780) and with a select group of parameters, by a flow cytometric reference method (FCM). Flagging rates were determined by comparing data from the DxH 800 and predicate LH 780 analyzer.

**Table 1:** The range of linearity of the new DxH 800 cellular analysis system and the LH 780

	Low limit		Upper limit	
		
Parameter	DxH 800	LH 780	DxH 800	LH 780
WBC (×10^9^/l)	0.000	0.000	400.000	400.00
RBC (×10^12^/l)	0.000	0.000	8.500	8.000
HGB (g/l)	0.00	0.00	255.0	250.0
MCV (fl)	50.00	50.00	150.00	200.00
PLT (10^9^/l)	0.0	0.0	3000.0	3000.00
NRBC (/100 WBC)	0.00	–	600.00	–
Retic (%)	0.000	–	30.000	–

WBC, white blood cell; PLT, platelet; NRBC, nucleated red blood cell.

## LH 780

The LH 780 is a fully automated random access analyzer with a throughput capability of 110 samples per hour (CBC/DIFF only). The analyzer generates a CBC, with a corrected WBC in the presence of interference, a WBC differential, a reticulocyte percentage and number, as well as a NRBC percentage and number. The WBC count is derived by an impedance technology similar to that of the DxH 800. Classification of WBCs is performed by threshold size discrimination (>35 fl). Interfering particles such as NRBCs, nonlysed red cells, giant PLTs, or PLT clumps, or fragmented cells which hover near this threshold potentially may be included in the WBC count. If any interfering particles are detected, the LH 780 generates a suspect message ‘cellular interference’ and will automatically correct the WBC count if necessary. Enumeration of the NRBC count is performed when particles are detected in the NRBC signature positions of both the WBC and the differential plots.

## Manual RBC indices correction

The standard laboratory method involved centrifugation of the sample and preparation of a concentrated red blood cell suspension, free of WBCs. The following calculations were applied; the corrected RBC count was determined by subtraction of the WBC count from the original RBC number of the neat sample. A corrected Hct was determined by multiplying the true MCV, derived from the RBC suspension, by the corrected RBC and dividing by 10. Finally, a corrected Hb was calculated by multiplying the true MCHC, derived from the RBC suspension, by the corrected Hct.

## Daily operations

Daily checks were performed each day as part of routine startup procedures. Startup was performed to ensure that the fluidics lines were purged of cleaning agent, and that the background counts, voltage readings, and reagent dates were within acceptable limits prior to data collection. Quality Control on all instruments was run daily to ensure that all instruments were operating within acceptable limits. Following daily operation, shutdown was performed ensuring appropriate cleansing of the fluidic system.

## Coulter XL-MCL

The flow procedures were performed on a single laser four-color Beckman Coulter XL-MCL flow cytometer. Alignment and calibration checks were performed daily.

## FCM reference procedures

WBC, PLT, and NRBC enumerations by FCM were performed as previously described (Chin-Yee *et al.*, 2001, 2002; Kaplan, Johnson & Wolfe, 2001).

WBC counts were performed in duplicate by staining 100 μl of accurately pipetted whole blood with 5 μl of both CD45-PC5 and CD41-FITC for 15 min at room temperature, protected from the light. Samples with a WBC count of ≥50 × 10^9^/l were diluted with 5% albumin. Following this, red cells were lysed with ammonium chloride and 100 μl of Flow-Count (Beckman Coulter) was accurately pipetted to each tube. Samples were kept on ice until the time of analysis. The average of the two reference counts was used.

For PLT enumeration, specimens were mixed gently by inversion and 5 μl of whole blood added to 100 μl of PBS/BSA and incubated with 10 μl of CD41/CD61- FITC for 15 min at room temperature, protected from the light. Samples were diluted to 1 : 1000 and immediately analyzed on the XL-MCL with the stop count set at 50 000 red cells events and a minimum of 2000 PLTs. For PLT counts >500 × 10^9^/l, specimens were diluted in a larger volume of PBS/BSA to reduce potential coincidence.

NRBC enumeration involved the incubation of 25 μl of whole blood with 10 μl CD45-FITC for 15 min at room temperature, protected from the light. Following this, samples were mixed and incubated for 30 s with 250 μl of an acidic lysing solution containing propidium iodide. To quench this lysing solution excess, 500 μl of alkaline reagent was subsequently added. Samples were then incubated for 5 min at room temperature prior to running on the XL-MCL.

## Manual WBC differential

Two blood films were prepared from each sample and independently examined by different technologists. Each technologist performed a 200-cell differential count. The number of NRBCs was expressed per 100 WBCs.

## Criteria for flagging

Specimens that generated ‘P’ flags (partial aspiration) as well as non-numeric results were excluded from the analysis. Samples were examined by manual review to determine the number of NRBCs and if giant PLTs or PLT clumping was present, only samples found to have NRBCs by manual review were considered positive for NRBCs, all others were considered negative.

## Statistical methods

Correlation and regression analysis was performed using Microsoft Excel. An analysis of variance (anova) was performed on the WBC, PLT, and NRBC results to determine whether a significance difference between groups was present. If a significant difference was found, a Wilcoxon test for nonparametric data was used to determine accuracy. Significant *P* values were values below 0.05, values greater than this were not significantly different. Biases were calculated on the both flagged and unflagged data. When the flagged data was removed from the analysis the biases and percentage differences were reduced on WBC, PLT, and NRBC data. False positive and false negative rates for PLTs and NRBCs were determined on the LH 780 and DxH 800 and used to determine sensitivity (TP/[TP + FN]) and specificity (TN/[TN + FP]) of each analyzer *vs*. FCM. Receiver Operator Curves (ROCs) were generated for the DxH 800 NRBC data *vs*. FCM. The Area Under the Curve (AUC) was determined from the ROC analysis (Aulesa *et al.*, 2004). The higher the AUC number the better the performance, with 0.50 indicating random performance and 1.00 denoting perfect performance.

## Results

The goal of this study was to evaluate the new Beckman Coulter UniCel® DxH 800 cellular analysis system with particular focus on correlation of WBC, NRBC and PLT enumeration to a predicate analyzer, the Coulter® LH 780 and FCM reference methods.

## WBC

**Figure 1:**
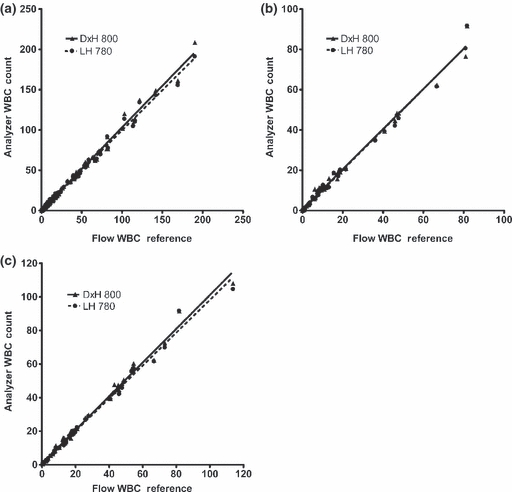
Leukocyte enumeration. (a) Leukocyte counts for all samples collected (*n* = 264) on the DxH 800 and LH 780 *vs*. flow cytometry (FCM). *r*^2^ Values for DxH 800 *vs*. LH 780 and DxH 800 *vs*. FCM were 0.998 and 0.994, respectively. (b) Leukocyte counts in the presence of cellular interference on the DxH 800 compared to the LH 780 and FCM. *r*^2^ Values for DxH 800 *vs*. LH 780 and DxH 800 *vs*. FCM were 0.996 and 0.990, respectively. (c) Leukocyte counts in the presence of nucleated red blood cells (manually confirmed) on the DxH 800 compared to LH 780 and FCM. *r*^2^ Values for DxH 800 *vs*. FCM and LH 780 *vs*. FCM were 0.998 and 0.992, respectively.

Samples were accrued in the WBC range of 0.1 to 190.4 × 10^9^/l (*n* = 247) and were examined to determine the precision and accuracy of the new DxH 800 analyzer. The DxH 800 WBC counts were correlated with the LH 780 and FCM during the study period. Lines of best fit were generated for all the data (Figure 1a) confirming that the new DxH 800 demonstrates extremely good correlation with the predicate reference LH 780 analyzer and the reference FCM (*r*^2^ = 0.998 and 0.994, respectively). Analysis of the WBC counts for accuracy showed there was no significant difference between the DxH 800 and the LH 780 or FCM (*P* = 0.918, anova). The DxH 800 showed no statistical difference between results when compared to both the LH 780 (*P* = 0.762) and FCM (*P* = 0.693) with no significant bias in leukocyte count, with or without the samples flagged for review removed (Table 2).

**Table 2:** Accuracy *P* values, bias (mean difference), and % mean difference for the DxH 800 cellular analysis system *vs*. the LH 780 and flow cytometry (FCM)

		DxH 800 *vs*. LH 780				DxH 800 *vs*. FCM			
Parameter	Subset	*P* value	*P* value(w/o flags)	Bias	% Diff	*P* value	*P* value(w/o flags)	Bias	% Diff
WBC	None	0.762	0.858	0.51	2	0.693	0.754	0.59	6
WBC	Cellular Interference	0.782	0.799	0.42	4	0.654	0.642	0.57	8
WBC	NRBC	0.879	0.931	0.69	3	0.736	0.762	0.73	6
PLT	None	0.716	0.625	−8	−7	0.510	0.657	−16	−9
PLT	<50 × 10^9^/l		0.833	0.24	24		0.831	−0.07	−7
PLT	Giant Platelets	0.784	0.773	−27	−4	0.276	0.640	−12	−17
PLT	Microcytosis	0.812	0.605	−12	−4	0.640	0.987	1	−5

WBC, white blood cell; PLT, platelet; NRBC, nucleated red blood cell.

A subset of the total number of samples (*n* = 46) were flagged for cellular interference, caused by the presence of either giant PLTs, PLT clumping, or NRBCs. The DxH 800 showed again high correlative results with the LH 780 (*r*^2^ = 0.996) and FCM (*r*^2^ = 0.990) in this subset (Figure 1b). These highly correlative results were also accurate (showing no statistical difference) when the DxH 800 was compared to the LH 780 (*P* = 0.782) and FCM (*P* = 0.654).

WBC count comparison of the DxH 800 in samples determined to have NRBCs revealed that the DxH 800 correlates well with both the LH 780 and FCM (Figure 1c, *r*^2^ = 0.998 and *r*^2^ = 0.992, respectively).

The DxH 800 detects and compensates well for the presence of interference to accurately enumerate WBCs without significant bias (see Table 2), The number of samples with flagged WBC values was similar between the DxH 800 and the LH 780 (4% and 3%, respectively).

## RBC parameters

A new feature of the DxH 800 is the automated correction of hemoglobin when the UWBC is ≥11 × 10^9^/l. Correction is also performed, as appropriate, on other parameters (RBC count, MCV, and RDW) when the UWBC is ≥140 × 10^9^/l. A set of eight samples had WBC counts ≥140 × 10^9^/l and were reviewed closely to determine the difference between the corrected counts on the DxH 800 and the standard laboratory method of handling extreme leukocytosis and the erroneous impact on red cell analysis. Results from the calculations detailed in the materials and methods section and from the DxH 800 are shown in Table 3. As can be seen, the hemoglobin, red blood cell count, and RDW values are comparable by both methods with a slightly lower MCV noted by the manual method.

**Table 3:** Evaluation of the DxH 800 automated RBC correction compared to manual RBC correction on high white blood cell (WBC) counts

	Red cell count (×10^12^/l)		Hemoglobin (g/l)		Mean cell volume (fl)		RDW (%)	
				
WBC (×10^9^/l)	DxH	Manual	DxH	Manual	DxH	Manual	DxH	Manual
161.2	2.45	2.42	84.0	81.0	99.69	97.5	14.56	14.7
101.8	3.23	3.16	96.9	100.0	100.56	98.3	17.26	18.1
114.3	3.12	2.90	104.1	102.0	109.16	102.4	15.01	15.5
149.1	4.00	3.84	122.0	122.0	92.20	88.3	16.29	16.6
137.1	4.08	3.79	123.4	119.0	98.06	92.0	14.08	14.1
120.1	3.72	3.47	112.0	114.0	99.03	91.4	13.48	13.6
108.1	2.74	2.73	77.8	83.0	86.00	83.8	15.58	16.2
208.6	3.35	3.33	110.8	108.0	98.16	95.5	15.58	15.1

## PLT

For this study, a FCM reference procedure was used to accurately validate PLT counts from the newly developed DxH 800 analyzer. Samples analyzed by FCM were in the PLT range of 1 to 1297 × 10^9^/l (*n* = 256). The DxH 800 showed a strong correlation with both the LH 780 and the FCM (*r*^2^ = 0.996 and *r*^2^ = 0.985, respectively). The DxH 800 gave accurate results when compared to both the LH 780 and the FCM (*P* = 0.804, anova) with a small, statistically insignificant negative bias (Table 2). The accuracy improved when samples that were flagged for review were excluded.

**Figure 2:**
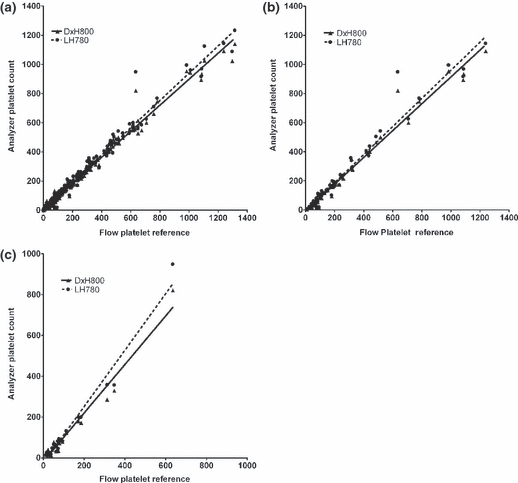
Platelet (PLT) enumeration. (a) PLT counts for all samples collected (*n* = 257) on the DxH 800 and LH 780 *vs*. flow cytometry (FCM). *r*^2^ Values for DxH 800 *vs*. LH 780 and DxH 800 *vs*. FCM were 0.996 and 0.985, respectively. (b) PLT counts in the presence of megathrombocytes (manually confirmed) on DxH 800 and LH 780 compared to the reference FCM. *r*^2^ Values for DxH 800 *vs*. LH 780 and DxH 800 *vs*. FCM were 0.996 and 0.974, respectively. (c) PLT counts in the presence of microcytosis (manually confirmed) on the DxH 800 *vs*. LH 780 compared to the reference FCM. *r*^2^ Values for DxH 800 *vs*. LH 780 and DxH 800 *vs*. FCM were 0.998 and 0.966, respectively.

A subset of all the samples (*n* = 57) contained megathrombocytes, a cellular characteristic that potentially could influence the PLT count. The DxH 800 showed strong correlation with both reference data sets again (LH 780 and FCM, Figure 2b) with *r*^2^ values of 0.996 and 0.974, respectively. A second subset of samples containing microcytic or fragmented RBCs (*n* = 28) were evaluated and again the DxH 800 analyzer demonstrated good correlation with the LH 780 and FCM data (Figure 2c) with *r*^2^ values of 0.998 and 0.966, respectively. Both of these PLT subsets showed no significant difference between groups (anova, *P* = 0.514 and *P* = 0.903, respectively). PLT accuracy data at <50, <20, <10 × 10^9^/l PLTs are shown in Table 4 show an impressive performance, particularly in the lower ranges. Of particular note, those samples with PLT counts <20 × 10^9^/l which showed disagreement, were flagged for review by the analyzer. The number of all samples with flagged PLT values was similar (10% on the DxH 800 and 9% on the LH 780) in this group of predominantly hematologically abnormal samples.

**Table 4:** Platelet (PLT) sensitivity and specificity values for the DxH 800 *vs*. flow cytometry

PLT count(×10^9^/l)	Sensitivity TP/(TP + FN)	Specificity TN/(TN + FP)	False negative
<10	92.3%	100%	1/13*
<20	83.3%	97.6%	5/30†
<50	96.6%	95.1%	2/58‡

* This single value has a value of 10.3.

† All samples were flagged for review by the DxH 800.

‡ These two samples only showed a small difference in PLT count.

## NRBC

**Figure 3:**
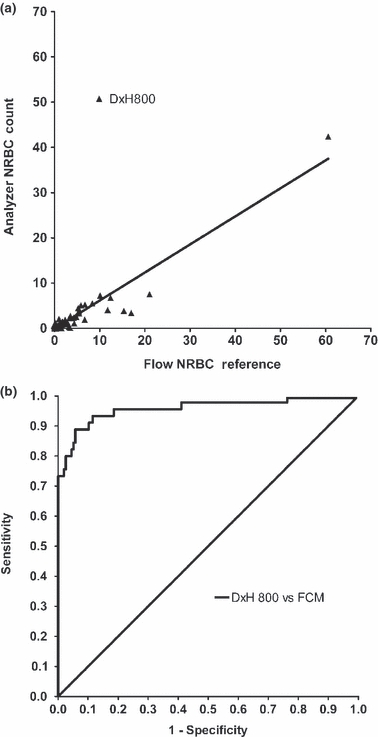
Nucleated red blood cell (NRBC) enumeration. (a) NRBC counts for all samples collected (*n* = 201) on the DxH 800 *vs*. flow cytometry (FCM). *r*^2^ Value for DxH 800 *vs*. FCM was 0.910. (b) Receiver Operator Curves for DxH 800 *vs*. FCM. Sensitivity for DxH 800 *vs*. FCM was 73% and specificity for DxH 800 *vs*. FCM was 100%.

A subset of samples were examined by manual review and FCM to determine the number of NRBCs in each (*n* = 246). Samples were in the range of 0 to 61 NRBCs/100 WBCs. The correct enumeration is clinically important as in the majority of situations it indicates stressed or disordered erythropoiesis and potential disease. A number of studies have repeatedly associated the presence of NRBCs with increased hospital mortality (Schwartz & Stansbury, 1954; Stachon *et al.*, 2002; Kho *et al.*, 2007). To determine the accuracy of NRBC enumeration we evaluated the new DxH 800 compared to a FCM reference method that very accurately enumerates NRBCs. The correlation coefficient between the DxH 800 and LH 780 was determined to be 0.819. However, comparison of the DxH 800 and LH 780 results to FCM (Figure 3a) showed a significant improvement in correlation with the DxH 800 (*r*^2^ = 0.910) when compared to the predicate LH 780 analyzer (*r*^2^ = 0.791). A significant difference (*P* < 0.001, anova) was found in the accuracy of the NRBC count between all three groups (DxH 800, LH 780, and FCM). Comparison of the DxH 800 *vs*. FCM counts showed no significant difference (*P* > 0.05, Dunn’s post-test) whereas the LH 780 showed a significant difference to the FCM counts (*P* < 0.05, Dunn’s post-test). This demonstrates improved NRBC enumeration by the DxH 800. ROC were generated for the DxH 800 *vs*. the reference FCM method. The AUC for the DxH 800 *vs*. FCM was determined to be 0.96 which shows a significant improvement over the predicate analyzer (AUC = 0.76). Further ROC analysis determined that NRBC enumeration on the DxH 800 *vs*. FCM had excellent specificity of 100% and sensitivity of 73% (Figure 3b).

The DxH 800 showed significant improvements over the LH 780 in false negative (FN) rate, false positive (FP) rate, sensitivity, specificity, and bias in the detection of NRBCs (shown in Table 5). Importantly, there was a significant decrease in false negative rate compared to the LH 780 (DxH 800, 6%*vs*. LH 780, 10%); furthermore, no false positives were detected on the DxH 800. The number of samples with the NRBC parameter flagged for review was reduced from 24% on the LH 780 to 14% on the DxH 800. Increased sensitivity and specificity, much improved correlation in particular in the range of 2–10 NRBCs (DxH 800 *vs*. FCM, *r*^2^ = 0.806 from LH 780 *vs*. FCM, *r*^2^ = 0.444), and decreased false negative/positive rate indicate that the DxH 800 is significantly better at detecting and enumerating NRBCs.

**Table 5:** Nucleated red blood cell false positive and false negative rates, sensitivity, specificity, and biases on the LH 780 and DxH 800

	LH 780	DxH 800
FP rate	3%	0%
FN rate	10%	6%
Sensitivity	56%	73%
Specificity	96%	100%
Bias	−0.29	−0.60

## Discussion

The CBC performed by current laboratory instruments is fast, reliable, and accurate on hematologically normal samples (Aulesa *et al.*, 2003; Bourner, Dhaliwal & Sumner, 2005). When all parameters are within normal ranges, the enumeration of cellular elements including a five-part differential are available for reporting in under a minute from presentation of the sample to the instrument. This process slows down considerably when the indices enumerated stray from normal values or there are abnormalities within the sample that cause flagging or interference, necessitating verification of the results prior to reporting. Cellular interference may influence the WBC count, giant or clumped PLTs may influence PLT enumeration leading to inaccurate clinical decision making, and the accurate detection and enumeration of NRBCs may indicate the presence of hematological or nonhematological disease (Schwartz & Stansbury, 1954; Harrison *et al.*, 2005; Segal *et al.*, 2005; Bodey, 2009).

Samples were accrued for determining the accuracy of the new DxH 800 in the ranges of 0.1 to 190.4 × 10^9^/l for WBC, 1 to 1297 × 10^9^/l for PLTs, and 0–61 NRBCs/100 WBCs. All samples were examined to confirm the accuracy and precision of the new DxH 800 analyzer compared to the predicate analyzer, LH 780, and Flow Cytometer Methods (FCMs) for total WBC, PLT and NRBC counts. WBC differentials were not compared in this study but may be the objective of future studies. The DxH 800 provides extremely precise and accurate PLT and WBC counts over an impressive reporting range considering that the samples were preselected based on abnormal hematological parameters. The DxH 800 also performs appropriate and accurate correction of red cell parameters in the presence of extreme leukocytosis (≥140 × 10^9^/l). WBC and PLT enumeration in the presence of interference results in a lower review rate and faster turn around times with the DxH 800, when compared to the LH 780. When compared to FCM, the DxH 800 shows no bias in WBC count and PLTs in the lower range; however, with higher PLT and NRBC counts, the DxH 800 shows a slight negative bias. The negative bias with high PLT values (which is not seen at PLT values <50 × 10^9^/l) may be attributed to the FCM reference. This reference method was originally developed for low PLTs numbers (<50 × 10^9^/l) and although a correction for higher PLT numbers (>500 × 10^9^/l) was used the FCM tends to overestimate the PLT value. This could account for the discordance seen in the clinically insignificant higher range between the DxH 800 and the FCM.

The DxH 800 cellular analysis system combines mechanical and technological improvements over previous analyzers resulting in 10 times more data collected per sample and translating into improved accuracy and precision. The demand for accurate and precise automated enumeration of WBC counts is driven by the demand placed on hematology laboratories for rapid turnaround times on samples with abnormal features. For WBC enumeration the most common features for interference that generate ‘flags’ are NRBCs, megathrombocytes, PLT clumping, and WBC counts in the lower range of linearity. Patients receiving active chemotherapy as cancer treatment require rapid and accurate results to allow for decisions on continuing treatment or to offer salvage therapy (Navarro *et al.*, 1998; Springer, von Ruecker & Dickerhoff, 1998; Sagmeister, Oec & Gmur, 1999; Cosler *et al.*, 2005; Nevo *et al.*, 2007; Oka *et al.*, 2007; Slichter, 2007; Dranitsaris *et al.*, 2008; Bodey, 2009).

Our laboratory evaluated the new DxH 800 and found that this new cellular analysis system is capable of providing accurate WBC counts in the presence of interfering particles and in the extreme low range (<0.5 × 10^9^/l). The DxH 800 does generate flags; however, the messages are more detailed than in previous analyzers and alert the operator to potential sample or enumeration issues. Manual review of these samples may still be necessary, depending on laboratory policy, in order that accurate results are communicated to the treating physician.

The heterogeneous morphology of NRBCs can lead to significant problems when enumerating WBC with impedance technology. The ISLH method for correction is dependent on manual review of suspect samples identified by NRBC flagging on a cellular analysis system. The number of NRBCs are then enumerated, expressed as the number present per 100 WBCs, and the WBC count is subsequently adjusted mathematically. The assumption that all NRBCs have been included within the WBC count may or may not hold true. NRBCs smaller than the WBC threshold will not have been included in the WBC count, whereas those greater than the threshold have the potential to have been. Manual correction for all the NRBCs seen by morphological review may lead to over correction of the WBC count. The DxH 800 uses advanced algorithms and the collection of ten times more data than previous analyzers to determine the ‘signature position’ without the need for a nuclear dye (increasing cost) or rerunning (increasing turn around time) as with other commercially available analyzers.

One of the most beneficial enhancements provided by the DxH 800, in our estimation, is the appropriate correction of red cell parameters in the presence of extreme leukocytosis. Automated and accurate correction of RBC parameters will allow for more rapid turn around times in patients with extreme leukocytosis, forgoing the need for manual manipulation and the potential for introduction of error.

The data in this study demonstrate that both test and comparator instruments (DxH 800 and LH 780, respectively) provide accurate PLT results and in the case of the DxH 800, when the counts are extremely low (<10 × 10^9^/l). A negative bias across the range of PLT values above 50 × 10^9^/l explains the low *P* values seen in Table 2. When PLTs in the critical range 0–50 × 10^9^/l were compared, there was no bias noted between the FCM and DxH 800 values. Cellular debris (and other substances) and large PLTs may be included or excluded in the PLT count and depending on their proportions the machine may generate a ‘flag’. Generation of the flag requires verification by another method (either manual chamber count or FCM), with the manual chamber count carrying a high coefficient of variation and FCM being impractical or unavailable for many laboratories. (Hanseler, Fehr & Keller, 1996; Harrison *et al.*, 2000). The DxH 800 boasts a significant increase in the amount of information (256 channels) collected on each sample. This combined with advances in algorithms and information sharing between other modules (potential interference detected in another parameter, e.g. microcytic RBC) help to reduce flagging and better enumerate PLTs at low levels. The accurate enumeration of low numbers of PLTs is important because thrombocytopenia may be a dose-limiting factor for cancer patients undergoing chemotherapy and also serves a trigger for PLT transfusion therapy (Navarro *et al.*, 1998).

The CSLI and ISLH guidelines have established the lower limit for detection of NRBCs to be 1 per 100 WBCs. We observed a small negative bias, (−0.6), in NRBC enumeration on the DxH 800 over the range from 0 to 60 NRBCs compared to FCM (FCM). It should be noted, however, that below 10 NRBCs the bias seen was very small. The ability to enumerate NRBCs at the lower limit is now available on the new DxH 800, representing a significant improvement.

The DxH 800 represents an improvement to previous analyzers with an extended range for key parameters, better sensitivity in the low range, as well as the accurate detection and enumeration of NRBCs at counts >1/100 WBCs. The reduction in flagging rate of the NRBC parameter should translate into reduced numbers of technically demanding and time-consuming blood film reviews. The automatic correction of RBC parameters in extreme leukocytosis eliminates the need for operator intervention and should significantly reduce the turn around time of severely abnormal results. These enhancements are afforded without the requirement of rerunning the sample or additional reagents.
